# Exportin 4 DNA promoter methylation in liver fibrosis

**DOI:** 10.1371/journal.pone.0302786

**Published:** 2024-05-09

**Authors:** Ziyan Pan, Ali Bayoumi, Mayada Metwally, Jacob George, Mohammed Eslam

**Affiliations:** Westmead Institute for Medical Research, Storr Liver Centre, Westmead Hospital and University of Sydney, NSW, Australia; University of Navarra School of Medicine and Center for Applied Medical Research (CIMA), SPAIN

## Abstract

A role for exportin 4 (XPO4) in the pathogenesis of liver fibrosis was recently identified. We sought to determine changes in hepatic XPO4 promoter methylation levels during liver fibrosis. The quantitative real-time RT-PCR technique was used to quantify the mRNA level of XPO4. Additionally, pyrosequencing was utilized to assess the promoter methylation status of XPO4. The methylation rate of the XPO4 promoter was significantly increased with fibrosis in human and mouse models, while XPO4 mRNA expression negatively correlated with methylation of its promoter. DNA methyltransferases (DNMTs) levels (enzymes that drive DNA methylation) were upregulated in patients with liver fibrosis compared to healthy controls and in hepatic stellate cells upon transforming growth factor beta (TGFβ) stimulation. The DNA methylation inhibitor 5-Aza or specific siRNAs for these DNMTs led to restoration of XPO4 expression. The process of DNA methylation plays a crucial role in the repression of XPO4 transcription in the context of liver fibrosis development.

## Introduction

Fibrosis is a progressive and potentially fatal process that can impact any organ [1]. Hepatic fibrosis is a key feature of chronic liver disease and a major contributor to all its adverse outcomes [2,3]. The development of chronic liver fibrosis is attributed to the activation of wound-healing mechanisms in response to liver damage, leading to structural changes in the hepatic parenchyma and vasculature, declines in liver functional reserve, cirrhosis, and the development of hepatocellular cancer [1,4].

Epigenetic modifications to DNA regulate whether genes are turned on or off and could represent a molecular link between the genetic background, environmental factors, and complex diseases. There is an increasing body of research indicating that epigenetic variables, particularly DNA methylation, play a significant role in the development of liver fibrosis [5,6]. The occurrence of atypical DNA methylation patterns including cytosines at CpG sites has the potential to initiate gene hypermethylation leading to altered gene transcription. This phenomenon has been shown in relation to many genes associated with liver fibrosis [5,6].

Exportin 4 (XPO4) is a member of the importin β family and serves as a nuclear transport receptor that facilitates bidirectional transport. The association between liver fibrosis and decreased hepatic expression of XPO4 has been demonstrated to occur via transforming growth factor-β (TGF-β)/SMAD3 signaling [7]. The decreased expression of XPO4 is also modified by a duplication in XPO4 copy number variation (CNV) [7].

According to recent research, there is evidence of a rise in the DNA methylation level of the XPO4 promoter in peripheral blood mononuclear cells (PBMCs) when chronic hepatitis B progresses to cirrhosis and hepatocellular carcinoma (HCC) [8]. However, the roles of XPO4 DNA methylation in fibrotic diseases, specifically liver fibrosis, remains speculative. Here, we explored the role of DNA methylation as an XPO4 gene regulatory element during liver fibrosis.

## Methods

### Human samples

The study included the quantification of DNA methylation levels in liver tissue samples from 20 people. The liver tissues were taken at surgical resection and stored at -80°C till used. The sample group consisted of 10 individuals with metabolic dysfunction-associated fatty liver disease (MAFLD)-related fibrosis, while the control group consisted of 10 normal individuals without this condition. Subjects with evidence of secondary causes of steatosis or alternative diagnoses were excluded, including alcohol abuse (men, >30 g/day; women, >20 g/day), total parenteral nutrition, chronic viral hepatitis (hepatitis B and hepatitis C), and drug-induced liver injury. The methodology for DNA methylation measurement has been previously published [9,10].

### Animal models

Three distinct mouse models of hepatic fibrosis were used in accordance with previously documented methodologies [7]. Following the conclusion of the experiments, the mice underwent weighing and were subsequently euthanized using ketamine (100 mg/kg body weight) and xylazine (20 mg/kg body weight) in normal saline via intraperitoneal injection. The mice were evaluated for the effectiveness of the anesthetic by measuring the absence of pain reflexes, then were euthanized through broking necks.

1. **Bile duct ligation (BDL)**: For fibrosis development, male C57BL/6 mice aged 6–8 weeks were subjected to BDL or sham surgery for a duration of 2 weeks.
2. **Carbon tetrachloride (CCl4)**: Male C57BL/6 mice, aged 6 to 8 weeks, were given intraperitoneal injections of CCl4 at a dosage of 1 ml per kilogram of body weight, twice a week for a duration of 4 weeks and harvested after 48 hours from the last dose of CCl4. The control group of mice was given a comparable amount of corn oil.
3. **Methionine and choline-deficient (MCD) diet:** Male C57BL/6 mice aged 6–8 weeks were administered either the MCD diet or a control diet enriched with methionine and choline.

The studies were carried out using a sample size of 6–8 mice per group, with the mice being provided unrestricted access to food and drink.

### Ethics

The patient samples were accessed for research purposes between January 2019—January 2020. The study obtained ethical clearance from the Sydney West Area Health Service and the Human Research Ethics Committee (HREC/17/WMEAD/433) at the University of Sydney. Written informed permission was obtained from all participants including genetic testing.

The Animal Ethics Committee of the Western Sydney Local Health District granted authorization for the conduct of animal experiments. The experimental protocols followed the Animal Experimentation criteria set out by the National Health and Medical Research Council (NHMRC) of Australia.

### Cell culture

The LX2 cell line, derived from immortalized human hepatic stellate cells, was cultivated in Dulbecco’s Modified Eagle Medium, containing 10% fetal bovine serum, 1% glutamine, 50U/mL penicillin and 50mg/mL.

### DNMTs silencing

The transfection procedure used Lipofectamine RNAiMAX (Invitrogen) in accordance with the manufacturer’s instructions. A specific Silencer siRNA (**S1 Table**) or Silencer Select negative control was utilized at a concentration of 50–75 nM. Following transfection for 24 hours, the cells were subjected to treatment with or without TGF-β1 (2.5 ug/ml) for a duration of 24 hours, subsequently, the cells were harvested. The assessment of transfection efficiency was conducted using real-time PCR.

## Pyrosequencing

### Bisulfite treatment of gDNA

A quantity of 500 ng of genomic DNAwas treated with bisulfite using the EZ DNA Methylation kit, produced by Zymo Research, Inc. CA. The DNA was subsequently purified using the manufacturer’s recommended procedure and then rinsed to a final volume of 46 μL.

### PCR

PCRs were conducted using 1 μL of DNA that had been treated with bisulfite, along with 0.2 μM of each primer. To purify the final PCR product, a primer was selected and then tagged with biotin. The primer was subjected to high-performance liquid chromatography (HPLC) purification. Following purification, sepharose beads were used to further purify the PCR product.

Human sequence:

YGTTTTGAGGAGGGGAAAGYGGGAAGAGYGATTTTAAGTTTAGGAAATTTTGTAYGGY GTTGGAGYGTGG

Mouse Sequence:

TTYGTTTAYGGATGTTTTTTTTTTATAGYGGAATGAATYGYGGAGYGGGAGG

### Pyrosequencing

The PCR product was affixed to Streptavidin Sepharose HP, a commercially available product produced by GE Healthcare Life Sciences. In accordance with the guidelines provided by the manufacturer, the immobilized PCR products underwent a series of steps including purification, washing, denaturation using a 0.2 μM NaOH solution, and subsequent washing using the Pyrosequencing Vacuum Prep Tool (Pyrosequencing, Qiagen). Subsequently, a concentration of 0.5 μM of the sequencing primer was used to facilitate the process of annealing the purified single-stranded PCR products. The 10 μL PCR products were subjected to Pyrosequencing using the PSQ96 HS System, a technique developed by Qiagen. The sequencing protocol was executed in accordance with the guidelines provided by the manufacturer. The evaluation of the methylation status of each sample was conducted using QCpG software, a specialized program created by Qiagen. The calculation of the mean methylation level included taking into account the methylation levels of all CpG sites that were analyzed within the specific area of interest for each gene. In order to determine if the input DNA underwent partial bisulfite conversion, non-CpG cytosines were included as internal controls throughout all experimental protocols. Furthermore, as per the experimental methodology, a set of DNA samples including both unmethylated and methylated regions are included as control specimens in each PCR. The experimental procedure included the combination of unmethylated control DNA with in vitro methylated DNA at various proportions, namely 0%, 5%, 10%, 25%, 50%, 75%, and 100%. Subsequently, the process of bisulfite modification, PCR, and pyrosequencing analysis was conducted to assess the occurrence of PCR bias.

### Determination of XPO4 CNV

The genotyping of the copy number variation (CNV) in the XPO4 13q12.11 region was conducted using the TaqMan CNV real-time, quantitative PCR assays, especially using Assay Hs03857719_cn. The determination of copy number was conducted using the CopyCaller™ software (version 2.0; Applied Biosystems), following the established methodology described in the literature [7].

### Real‐time PCR analysis

RNA was isolated from LX-2 cells using the RNeasy kit (Qiagen) in accordance with the manufacturer’s instructions. The experiment included the use of 1000 ng of RNA which was subjected to reverse transcription to generate complementary DNA (cDNA). This process was carried out using the qScript cDNA SuperMix (catalog number 95048–500, Quantabio). For real-time PCR analysis, a 96-well plate was used to introduce a volume of three microliters of diluted complementary DNA, in conjunction with SYBR Select Master Mix (Applied Biosystems, Thermo Fisher Scientific). Taqman Probes, together with forward and reverse primers, were carefully designed for each specific gene of interest. The aforementioned components were used at a concentration of 10 picomoles per microliter. GAPDH was used as the reference gene for the purpose of normalization.

### Statistical analysis

The statistical analyses were conducted using Graphpad Prism 8 software. The data are provided in as means with their corresponding Standard Error of the Mean (SEM). For comparisons between two groups, the Student’s t-tests was used. A one-way analysis of variance (ANOVA) was used to compare several conditions. Statistical significance was operationally defined in this study as *P < 0.05, **P < 0.01, and ***P < 0.001.

## Results

### XPO4 hypermethylation in hepatic fibrosis in human and mice

**Fig 1:**
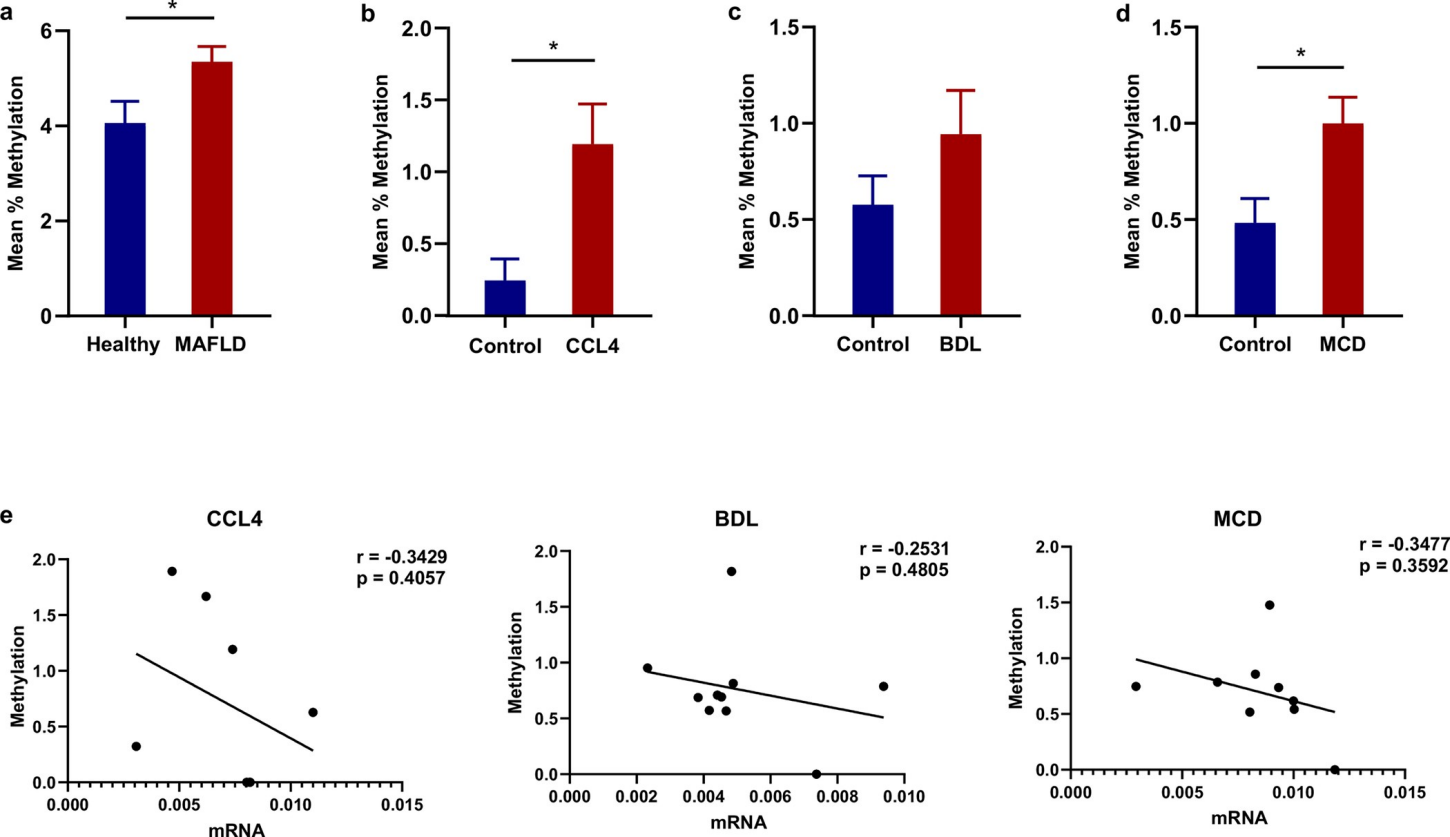
XPO4 expression is silenced through DNA hypermethylation in fibrosis. **a**) Increased hepatic XPO4 DNA methylation in patients with MAFLD and **b-d**) in three mouse models CCl4, BDL and MCD. **e**) Hepatic Xpo4 mRNA expression is negatively correlated with Xpo4 DNA methylation in the mouse models. The results are presented as the mean value ± SEM. The statistical significance of the observed differences between groups was assessed by using the unpaired two-sample Student’s t-test. The significance levels used in this study were denoted as follows: *P < 0.*05*, ** P<0.*01*, *** P<0.*001*.

Based on previous findings of persistent downregulation of XPO4 expression in the fibrotic liver [7], we hypothesised that epigenetic mechanisms might play a part in contributing to the phenotype. To evaluate whether DNA methylation is implicated, we analysed XPO4 promoter CpG island methylation in human liver from subjects with MAFLD-related fibrosis or healthy controls (n = 10, per group) using bisulphite pyrosequencing. This showed XPO4 hypermethylation in MAFLD liver, compared to controls (**Fig 1A**).

Similar findings were demonstrated in liver from three models of liver fibrosis in mice, namely CCl4, BDL and MCD. In each model, XPO4 methylation level was found to negatively correlate with XPO4 mRNA expression (**Fig 1B–1E**). In total, these data indicate that XPO4 transcription is in part regulated through DNA methylation.

### XPO4 is methylated by DNMTs in activated HSCs

**Fig 2:**
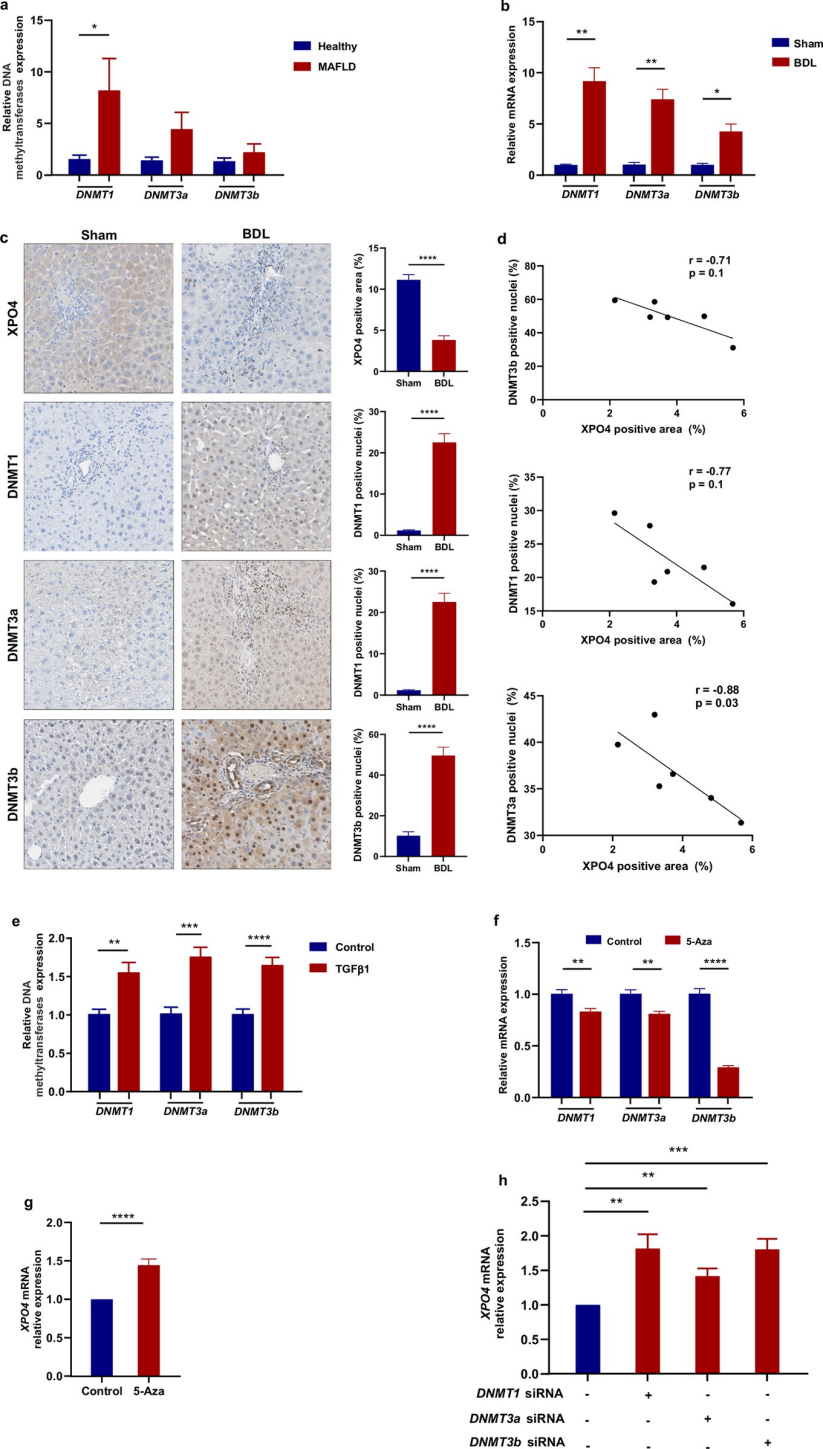
**a**) The hepatic mRNA expression of DNA methyltransferases (DNMTs), *DNMT1*, *DNMT3a*, and *DNMT3b*, the enzymes that result in DNA methylation is upregulated in patients with MAFLD-fibrosis compared to healthy group and **b)** in bile duct ligation (BDL) compared to sham. **c)** Representative expression pattern of XPO4, DNMT1, DNMT3a, and DNMT3b in livers from mice with bile duct ligation (BDL) and sham (n = 3 per group). Original magnification: 200×; and the intensity of expression was quantified digitally using ImageJ. **d)** Correlation between DNMTs and XPO4 expression was assessed by Spearman’s rank correlation. **e**) TGFβ1 increases DNMTs in LX-2 cells. **f)** 5-Aza decreases DNMTs mRNA expression in HSCs. **g**) 5-Aza increases XPO4 gene expression in HSCs. **h**) Specific siRNAs for these DNMTs in LX-2 cells led to increased *XPO4* expression. The results are presented as the mean value ± SEM. The statistical significance of the observed differences between groups was assessed by using the unpaired two-sample Student’s t-test. The significance levels used in this study were denoted as follows: *P < 0.*05*, ** P<0.*01*, *** P<0.*001*.

What induces XPO4 methylation in fibrosis is unknown. DNA methylation is mediated by DNA methyltransferases (DNMTs) [11]. TGFβ is the archetypal pro-fibrotic cytokine that is increased in liver fibrosis and is known to induce DNA methylation [12]. The repression of XPO4 upon hepatic stellate cell (HSC) stimulation with TGFB has recently been shown [7]. In order to ascertain the probable factors that initiate hypermethylation upstream, we conducted an investigation on the primary DNMTs. In this analysis, hepatic expression of DNMT1, DNMT3a, and DNMT3b was significantly increased in individuals with fibrosis as compared to a control group of healthy individuals (**Fig 2A**). Similarly, hepatic mRNA and protein expression of DNMT1, DNMT3a, and DNMT3b was significantly increased in BDL mouse model of fibrosis as compared to a control group by RT-PCR (**Fig 2B**) and immunohistochemistry (**Fig 2C**), respectively. The expression of DNMTs was found to be negatively correlated with XPO4 expression (**Fig 2D)**. Additionally, in LX2 cells (a human HSC cell line), TGFβ1 was confirmed to induce the expression of fibrotic markers (**S1 Fig**) and induced the expression of DNMT1, DNMT3a, and DNMT3b (**Fig 2E**).

To confirm that silencing of XPO4 expression is via methylation. We treated LX2 cells with the DNA methylation inhibitor 5-Aza. We confirmed that 5-Aza attenuates DNMTs expression, which was more profound for DNMT3B (**Fig 2F**). This led to increased XPO4 mRNA expression (**Fig 2G**). For further confirmation, we used specific siRNA for these DNMTs in LX-2 cells. The silencing efficacy was confirmed at mRNA and protein levels (**S2A and S2B Fig**). This again led to increased XPO4 mRNA (**Fig 2D**) and protein expression (**S2C Fig**).

### XPO4 CNV is not associated with XPO4 DNA methylation

**Fig 3:**
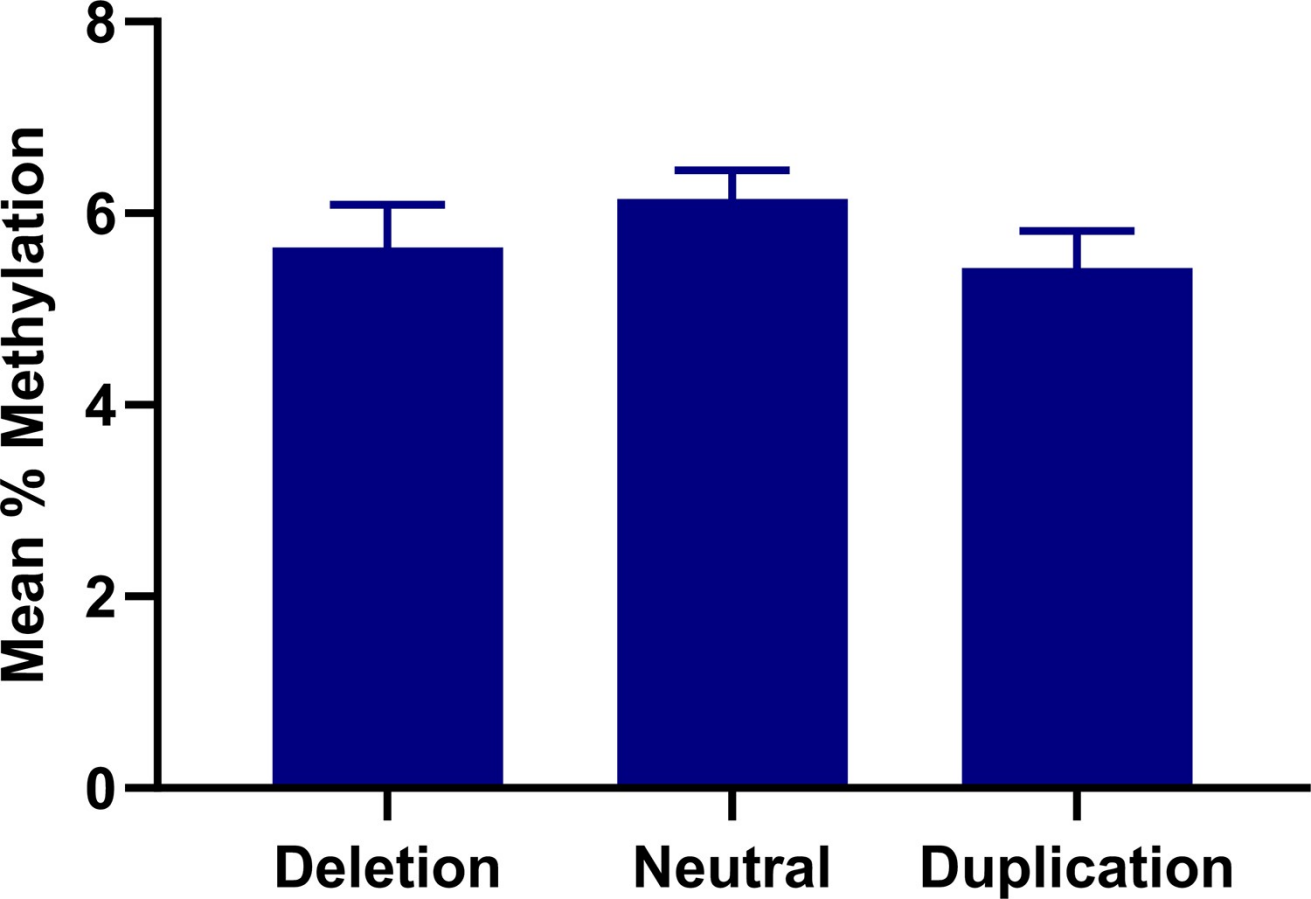
XPO4 CNV is not associated with XPO4 DNA methylation. The outcomes are presented in the form of the mean value plus or minus the SEM. The statistical significance of the observed differences between groups was determined by using the one-way ANOVA method.

Recent studies have shown an association between some CNVs and DNA methylation patterns that could mediate their impacts on gene regulation [13]. For example, a CNV in the XPO4 gene was found to be associated with lower hepatic XPO4 expression and increased fibrosis [7]. Therefore, we investigated if the XPO4 CNV does this via regulation of DNA methylation in a cohort of 75 patients with MAFLD related fibrosis. XPO4 CNV was not found to be associated with XPO4 DNA methylation (**Fig 3**). Collectively, CNV and DNA methylation, acting independently, are the main drivers of XPO4 downregulation in MAFLD-fibrosis.

## Discussion

We identified aberrant hypermethylation of XPO4 during hepatic fibrosis in humans and mice and an inverse correlation between XPO4 promoter methylation and its mRNA expression. This suggests that epigenetic silencing of XPO4 is one of the mechanisms modulating XPO4 expression during liver fibrosis.

Epigenetic regulatory mechanisms have the potential to function as a dynamic intermediary between genetic vulnerability and environmental signals to govern the clinical phenotype. Among them, DNA methylation stands out as the most extensively studied epigenetic alteration in humans, playing a pivotal role in the development of complex diseases [5]. Moreover, there is a growing body of research indicating that the pathophysiology of liver fibrosis is influenced by epigenetic factors, with DNA methylation playing a significant role [14]. Given the possible reversibility of epigenetic modifications, characterization of the epigenetic profile of genes implicated in liver fibrosis holds promise for the discovery of novel therapeutic targets [14].

A recent study revealed decreased XPO4 hepatic expression with advancement of liver fibrosis through regulation of transforming growth factor beta (TGF-β)/SMAD3/SMAD4 -mediated HSC activation [7]. In that study, XPO4 has preferential binding to SMAD3 compared to other members of the SMAD family. This leads to XPO4-mediated termination of nuclear SMAD3 signalling that is further modified by a duplication in the XPO4 CNV. The latter is associated with lower hepatic XPO4 expression and a greater extent of liver fibrosis [7]. We extend this work and identify another mechanism for XPO4 suppression during fibrosis via DNA hypermethylation. Notably, no correlation was discerned between XPO4 CNV and DNA methylation, implying that they are both independent mediators of XPO4 downregulation during liver fibrosis.

Fibrosis is characterised by an exaggerated wound healing response and the build-up of extracellular matrix proteins, namely type I and type II collagen [15,16]. Myofibroblasts (HSCs) play a role in the excess synthesis of extracellular matrix proteins in fibrotic tissues regardless of the underlying cause [17]. The process of DNA methylation and demethylation is an epigenetic modification characterized by its dynamic nature and heritability. Epigenetic modifications do not modify the DNA sequence, but rather induces alterations in gene expression, potentially leading to gene silencing [18]. Conversely, hypomethylation of DNA has been associated with heightened gene expression [19]. Approximately 70% of promoters in mammals are found to possess CpG islands [20]. Thus, it is plausible that DNA methylation plays a role in transcriptional processes and the development of fibrosis [21].

DNMTs are a class of enzymes that has significant importance in the intricate process of DNA methylation control. Regulation takes place by the transfer of a methyl group from S-adenosyl-L-methionine (SAM), a widely used methyl donor, to the 5-position of cytosine residues inside DNA [22]. Mammals possess three primary DNMTs, namely DNMT1, DNMT3a, and DNMT3b, which have the capacity to demonstrate synergistic interactions. DNMT1 has a predilection for a template that possesses hemi-methylation, hence facilitating the maintenance of pre-existing DNA methylation patterns. DNMT3A and DNMT3B are enzymatic proteins that belong to the category of de novo methyltransferases. These enzymes play a crucial role in the initiation of DNA methylation patterns on DNA molecules, regardless of their initial methylation status, by adding methyl groups to previously unmethylated or already methylated DNA regions [23,24].

Elevated levels of DNMTs have the potential to induce excessive methylation of the promoter region of the XPO4 gene, hence influencing gene expression. Our study provides mechanistic evidence supporting the notion that DNMTs exhibit upregulation in the liver tissues of patients with fibrosis associated with MAFLD. A similar trend was seen in human HSCs after activation with TGF-β. In contrast, the process of DNA methylation was impeded by the administration of 5-aza, a pharmacological agent known for its inhibitory effects on methylation [25], or by specific silencing of DNMTs. Our discovery establishes a connection between the epigenetic machinery and the activation of HSCs and so contributes to a more comprehensive understanding of the molecular processes implicated in liver fibrosis. Further investigation is necessary to explore the intricate underlying mechanisms concerning whether the modification of the DNA methylome is a consequence of the TGFβ–SMAD signaling cascade and to comprehend the precise manner in which XPO4 influences the impact on profibrogenic factors, such as TGFβ, on the DNA methylome.

To the best of our current understanding, this study represents the first investigation into the potential involvement of XPO4 methylation in the development of fibrosis. Our study is broadly consistent with a recent report that showed an increase in the methylation rate of the XPO4 promoter accompanied by a reduction in transcription of the XPO4 gene in peripheral blood mononuclear cells (PBMCs) in association with the progression of hepatitis B infection and hepatocellular carcinoma development [8]. Further research would be needed to explore the XPO4 methylation contribution to liver cancer development in patients with MAFLD. Additionally, given that the TGFβ–SMAD pathway contributes to progressive fibrosis in several different organs including liver, kidney, lung and heart [1,26,27], it would be interesting to explore whether the fibrosis-related XPO4 changes we identified are unique to liver or are common to other organs.

In conclusion, our results suggest that DNA hypermethylation is in part responsible for the previously reported downregulation of XPO4 expression during progressive liver fibrosis. In addition, both CNV and DNA methylation represent independent mechanisms that regulate XPO4 gene transcription.

## Supporting information

S1 Fig Test of TGFβ used in the experiment. *ACTA2*, *COL1A1*, *TGFβ1* mRNA expression was assessed by RT-PCR and normalized to *GAPDH* in response to TGFβ stimulation for 24h in LX2 cells, n = 6 per group. The data were analyzed using the unpaired two-sample Student’s t-test and is represented as means ± sem. ** P <0.01, *** P <0.001.
(TIF)

S2 Fig Test of siRNAs used in the experiment. LX2 were transfected with scrambled or DNMT1, DNMT3a, and DNMT3b siRNA for 48 hours and their relative mRNA expression was assessed by **a)** RT-PCR and **b,c)** protein level by Western and normalized to GAPDH. The data were analyzed by one-way ANOVA; multiple comparisons were corrected by Bonferroni correction and represented by means ± sem. ** P <0.01, *** P <0.001.
(TIF)

S1 Table List of siRNAs and negative control used. (DOCX)
