# Supplementary figures and images for: Exportin 4 DNA promoter methylation in liver fibrosis
Source: PLoS One. 2024 May 9;19(5):e0302786. doi: 10.1371/journal.pone.0302786 (PMC11081319; doi:10.1371/journal.pone.0302786)

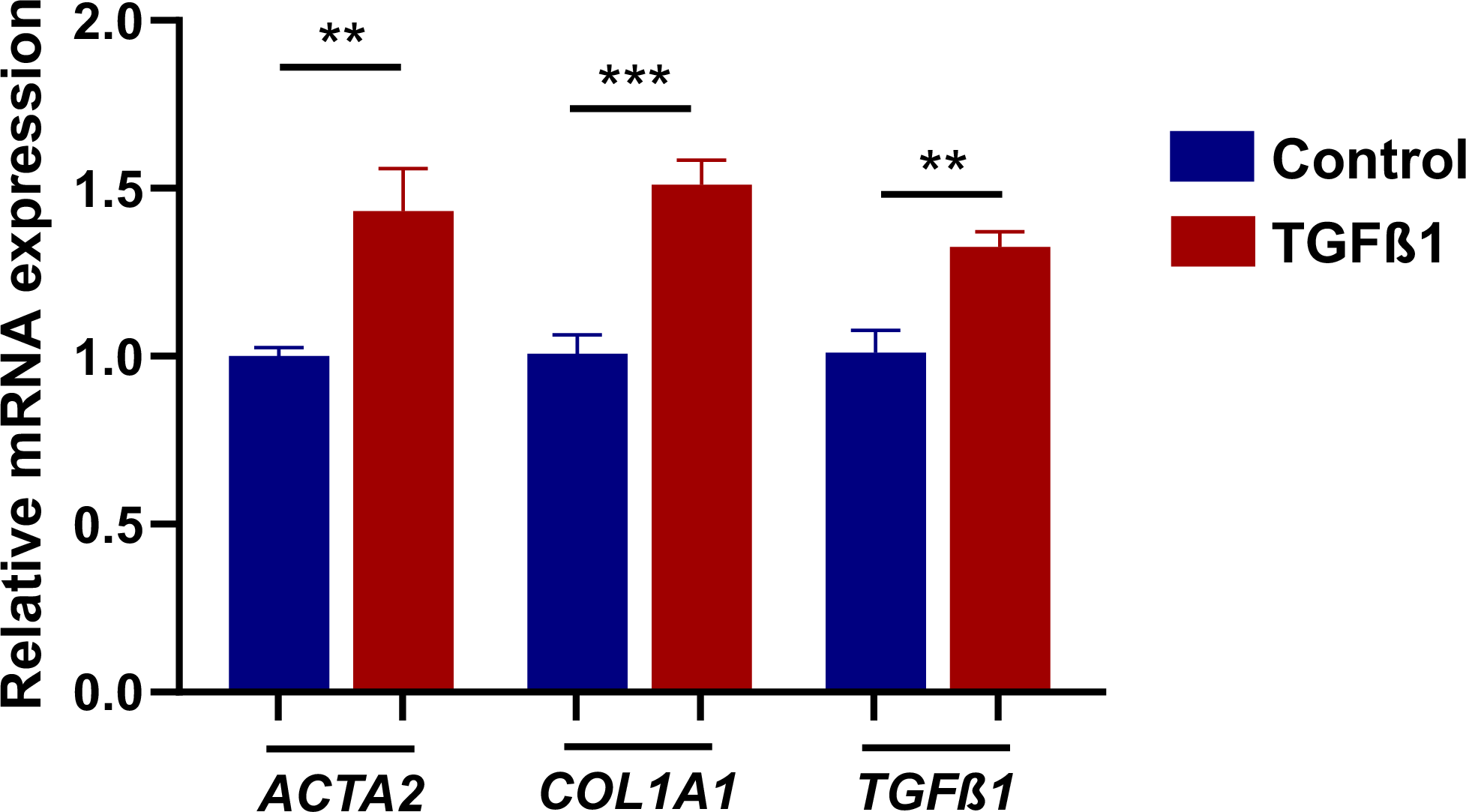

Supplement: S1 Fig — ACTA2, COL1A1, TGFβ1 mRNA expression was assessed by RT-PCR and normalized to GAPDH in response to TGFβ stimulation for 24h in LX2 cells, n = 6 per group. The data were analyzed using the unpaired two-sample Student’s t-test and is represented as means ± sem. ** P <0.01, *** P <0.001. (TIF) [file pone.0302786.s001.tif]

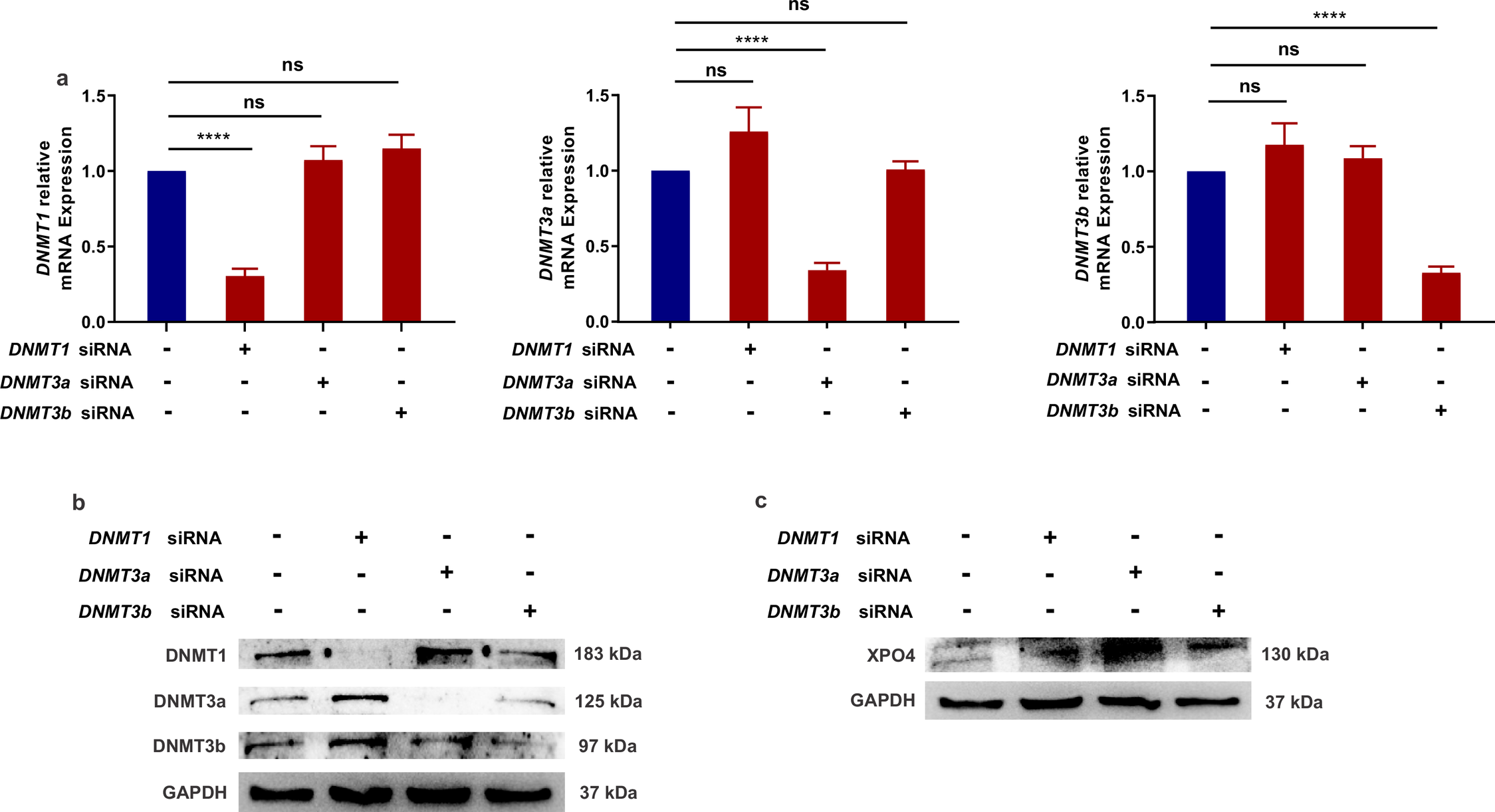

Supplement: S2 Fig — LX2 were transfected with scrambled or DNMT1, DNMT3a, and DNMT3b siRNA for 48 hours and their relative mRNA expression was assessed by a) RT-PCR and b,c) protein level by Western and normalized to GAPDH. The data were analyzed by one-way ANOVA; multiple comparisons were corrected by Bonferroni correction and represented by means ± sem. ** P <0.01, *** P <0.001. (TIF) [file pone.0302786.s002.tif]
