# Supplementary material for: Exportin 4 DNA promoter methylation in liver fibrosis
Source: PLoS One. 2024 May 9;19(5):e0302786. doi: 10.1371/journal.pone.0302786 (PMC11081319; doi:10.1371/journal.pone.0302786)
Supplement: S1 Table — (DOCX) [file pone.0302786.s003.docx]

**S1 Table: List of siRNAs and negative control used.**

| **SiRNA name** | **Cat no** | **company** |
| --- | --- | --- |
| ON-TARGETplus Human DNMT1 (1786) siRNA-SMARTpool | L-004605-00-0005 | Dharmacon |
| ON-TARGETplus Human DNMT3A (1788) siRNA-SMARTpool | L-006672-01-0005 | Dharmacon |
| ON-TARGETplus Human DNMT3B (1789) siRNA-SMARTpool | L-006395-00-0005 | Dharmacon |
| ON-TARGETplus Non-targeting Pool | D-001810-10-05 | Dharmacon |
